# Effect of Mixed Probiotics on Ovalbumin-Induced Atopic Dermatitis in Juvenile Mice

**DOI:** 10.1155/2024/7172386

**Published:** 2024-03-31

**Authors:** Jinli Huang, Xingzhi Wang, Qiuhong Li, Panpan Zhang, Zenghui Jing, Juan Zhang, Hui Su, Xin Sun

**Affiliations:** ^1^Department of Pediatrics, Xijing Hospital, The Fourth Military Medical University, Xi'an 710032, China; ^2^Department of Geriatrics, Xijing Hospital, The Fourth Military Medical University, Xi'an 710032, China

## Abstract

Atopic dermatitis is one of the most common dermatologic problems, especially in children. Given the ability of symbiotic microorganisms in modulating the immune system, probiotics administration has been studied in previous research in the management of atopic dermatitis. However, there are conflicting results between studies. In this study, we aimed to assess the effectiveness of mixed probiotics as a treatment option for atopic dermatitis induced by ovalbumin. BALB/c juvenile mice were classified and divided into the ovalbumin group, mixed probiotic group (ovalbumin + LK), and control group. Except for the control group, all mice were sensitized with ovalbumin to establish a model of atopic dermatitis. The mixed probiotics were given by gavage for 14 days. Mice body weight, skin lesions, skin inflammation, ovalbumin-specific Ig, the number of Treg and CD103^+^DC, and the expression level of PD-1/PD-L1 were examined. The results showed that mixed probiotics can improve body weight and alleviate skin symptoms. Mixed probiotics reduced serum Th2 inflammatory factors, eosinophils, mast cell degranulation, mast cell count, and the expression of ovalbumin-specific immunoglobulin E/G1 and increased the anti-inflammatory cytokine interleukin-10, Treg cells, CD103^+^DC cells, and the expression level of PD-1/PD-L1. These findings suggest that mixed probiotics could be a viable treatment option for atopic dermatitis and provide insight into the underlying mechanisms involved.

## 1. Introduction

Atopic dermatitis (AD) is a common inflammatory skin disorder characterized by recurrent eczematous lesions and intense itch [1, 2]. Over the past few decades, there has been an increase in both the prevalence and incidence of AD [2]. According to the Global Burden of Disease Study, AD affects 15%–20% of children and up to 10% of adults, ranking 15th among the most common nonfatal dermatitis [3]. The clinical manifestation of pruritus can significantly impact the health and quality of life of AD patients. While current treatments for AD, such as topical steroids and immunosuppressants, provide symptomatic relief, they do not offer a complete cure. Therefore, there is an urgent need for the development of a more effective treatment method for AD. The early human microbiome and immune cells had a significant impact on the development of the immune system. Disrupting the microbiota's growth and maturation during early childhood can have adverse effects on the immune system, potentially leading to allergies [4, 5]. Proper regulation of the immune system is crucial in treating AD [6]. The imbalance between T-helper 2 (Th2) and T-helper 1 (Th1) cytokines in AD causes changes in cell-mediated immune responses and promotes immunoglobulin E (IgE)-mediated hypersensitivity [7].

The interaction pathway of programmed cell death 1 and its ligand 1 (PD-1/PD-L1) can generate inhibitory signals that control T lymphocyte activation, tolerance, and immune-mediated tissue damage [8]. Animal experiments found that the PD-1/PD-L1 pathway plays a key role in the early formation and later maintenance of immune tolerance [9], however, its role in AD has not been elucidated.

Probiotics are live microorganisms that have been shown to provide health benefits when consumed in sufficient quantities [10]. Current studies have found that *Lactobacillus*, *Bifidobacteria (B)* strains, *Lactobacillus* (*L*) *rhamnosus*, and *Lactobacillus GG* et al. have the potential to alleviate AD, reduce atopic dermatitis index score, and reduce the risk of developing AD. Beneficial bacteria such as *Bifidobacterium* and *Lactobacillus* are in shortage in AD patients. Probiotics promote the synthesis of nutrients such as amino acids and vitamins in the host and increase the content of short-chain fatty acids (SCFAs) in the intestinal lumen. Especially, SCFA including acetate, propionate, and butyrate leads to an intestinal environment with a low pH value to inhibit the growth of pathogens. In addition, probiotics compete with the pathogens, including for nutrient substrates and ecological niches, and these interactions help to inhibit the overproliferation of pathogens in the gut [11]. Moreover, probiotics may alleviate the clinical manifestations of AD by influencing immune responses. *Bifidobacterium* is known to stimulate Peyer's patches to induce IgA production and maintain the integrity of the gut barrier. Administration of *Lactobacillus GG* and *Saccharomyces boulardii* affects the cytokine release and mucosal milieu, and this increases IgA production in the intestine [12]. The research found *L. plantarum LM1004* and *B. animalis subspecies lactis BB-12* significantly improved the AD-like symptoms, decreased Th2 and Th17 cell transcription factor levels, and increased the transcription factors of Treg and Th1 cells [13, 14]. *L. paracasei KBL382* regulated immune balance via increasing the expression of IL-10 and transforming growth factor-*β* and enhancing the differentiation of CD4^+^CD25^+^ Foxp3^+^ Treg in mesenteric lymph nodes [15]. *L. sakei WIKIM30* enhanced Treg differentiation in mesenteric lymph nodes via inducing DCs tolerance and ameliorated AD-like skin lesions [16]. In both animal models, whether healthy or diseased, human clinical trials have reported immune stimulation in hosts who consume probiotics [17, 18]. Probiotics can affect the host's immune response through beneficial modifications of the gut microbiota. However, the exact mechanisms behind this action are still not fully understood [19].

Previous studies have shown that mixed probiotics can reduce the occurrence of asthma and food allergies [20, 21]. Furthermore, some studies have indicated that probiotics can also improve the symptoms of AD in children [22]. However, the mechanism of how mixed probiotics treat AD and their effects are not yet clear. Therefore, this study aims to investigate the inflammatory and immunomodulatory effects of mixed probiotics in OVA-induced AD mice and explore the underlying mechanism of their effects.

## 2. Materials and Methods

### 2.1. Preparation of Probiotics

Probiotics (lyophilized powder, at a concentration of 5 × 10^10^ CFU live total bacteria/g, supplied by Ningbo Lvkang Rehabilitation Technology Co., Ltd., China) containing six bacterial strains (*Lactobacillus gasseri* LK001, 40%; *Lactobacillus salivarius* LK002, 20%; *Lactobacillus johnsonii* LK003, 15%; *Lactobacillus paracasei* LK004, 5%; *Lactobacillus reuteri* LK005, 5%; and *Bifidobacterium animalis* LK011, 15%) without additives were used as therapeutic agents in the study.

In accordance with our previous article, each strain was obtained from the intestinal tract of healthy newborns [21]. Lyophilized powders of probiotic mixture were prepared by using a high-density culture system (6kl microbial FV system designed for GTW (Shanghai) Fluid Handling System) provided by ProMD Biotech Company Limited. The powders were stored at −4°C and stability tests have shown that the cell viability remains intact for up to 3 months at room temperature. Each probiotic strain in the mixture has been certified for food use by the National Health Commission of the People's Republic of China and is identified and preserved by the China Industrial Culture Collection to ensure its safety and purity.

### 2.2. Mice

BALB/c mice at 15 days of gestational age were purchased from the Laboratory Animal Center of the Fourth Military Medical University and housed at a constant room temperature of 21–25°C, humidity of 50%–65%, light for 12 h/d, adaptive rearing until natural delivery, and a standard diet and water were provided ad libitum. All animal experimental procedures were approved by the Ethics Committee of the Fourth Military Medical University.

### 2.3. Experimental Design

**Figure 1:**
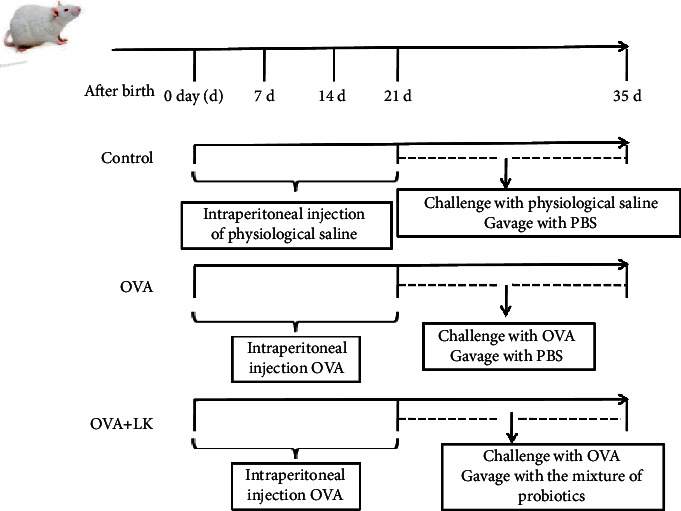
The animal experimental protocol.

Figure 1 depicts the animal experimental protocol. The study involved juvenile mice that were divided into three groups: the ovalbumin (OVA) group, the OVA + LK group that received the probiotic treatment with OVA sensitization and challenge, and the control group.

Mice in OVA and OVA + LK groups were sensitized intraperitoneally with OVA (10 *μ*g per mouse; Sigma-Aldrich, St. Louis, MO, U.S.A.) and 800 *μ*g of AL(OH)3 (Tianjin Tianshili Chemical Reagent Co, Ltd, Tianjin, China) dissolved in PBS, and each mouse was injected with 50 *μ*l on days 0, 7, and 14. The control group was given 50 *μ*l of PBS at the same time point. For the challenge of AD, the mice in control, OVA, and OVA + LK groups were first given a haircut on their back and then an 8% sodium sulfite solution was applied to the irritated area. After 5 minutes, the removed hair was wiped off with a clean gauze. From days 21–35, gauze strips moistened in 1% OVA solution were placed on the skin stimulation site and changed every two days in OVA and OVA + LK groups. The control group was given saline-moistened gauze strips for the challenge. The OVA + LK group was gavaged with 0.2 ml of mixed probiotics (5 × 10^10^ CFU/ml, dissolved in PBS) per day from day 21 to day 35. On the other hand, both the OVA and control groups were gavaged with 0.2 ml of PBS per day from day 21 to day 35. Before the end of the experiment, the body weight of the mice was weighed for the last time, and the skin tissue treated with normal saline or 1% OVA was taken for histopathological detection. After the mice were anesthetized with pentobarbital sodium, the eyeballs were removed, and blood was taken for the detection of inflammation indicators and OVA-specific antibodies. After dissecting the mice, the skin-draining lymph nodes were taken for flow cytometry analysis to detect the changes in the PD-1/PD-L1 signaling pathway.

### 2.4. Skin Lesions and Weight

Before the mice were sacrificed, the body weight in each group was measured. To evaluate skin lesions, the severity of dermatitis was determined by using the scoring system proposed by Matsui et al. [23]. The lesions on the back (8 cm^2^ of skin) and each ear (1 cm^2^ of skin) were scored. The dermatitis score on a scale of 0 (no symptoms), 1 (less than 1/3 of the skin area), or 2 (1/3 and more of the skin area),across five symptoms: 1) redness/scratch marks, 2) edema/lichenification/thickening, 3) hemorrhage/scabbing, 4) erosion, and 5) desquamation.

### 2.5. Cytokines and Inflammatory Cells

Blood samples were collected from mice and allowed to stand for 2 hours before being centrifuged at 3000 rpm for 15 minutes to obtain the serum. Interleukin 4 (IL-4), Interleukin 5 (IL-5), Interleukin 13 (IL-13), Interleukin 10 (IL-10), and mast cell protease-1 (MCPT-1) were measured using ELISA kits (RD System, Boston, MA, USA) as per the manufacturer's instructions. To determine the number of inflammatory cells, 5 *μ*l of blood was used to prepare blood smears stained with Wright–Giemsa, and the eosinophil count was determined under a light microscope.

### 2.6. OVA-Specific Antibodies

ELISA kits (RD system, Boston, MA, USA) were utilized to measure the concentrations of OVA-IgGl and IgE, in accordance with the manufacturer's instructions.

### 2.7. Flow Cytometry

Single-cell suspension isolated from skin-draining lymph nodes (SLNs) was prepared and stained for fluorescence-activated cell sorting (FACS) analyses [24]. Cells were stained with CD103-FITC, CD11c-PE, CD40-APC, CD80-eflour710, and PD-L1-PE-Cyanine7 to detect DCs. Cells were stained for surface markers using CD4-FITC, CD25-PE Cy7, and PD1-APC (eBioscience, Carlsbad, CA, USA). According to the manufacturer's instructions, the intracellular marker Foxp3-PE was stained by using a fixation/permeabilization solution (BioLegend). Data were analyzed by using the BD FACSCanto system (BD Biosciences, San Jose, CA, USA) and FlowJo 10.7 software (Tree Star Inc., Ashland, OR).

### 2.8. Histopathology

Skin infiltrated by normal saline in the control group and skin infiltrated by 1% OVA in the OVA and OVA + LK groups were mainly taken from erythema, and three parts were taken from each mouse. The tissue parts of mice in the three groups were the same. Mouse skin was fixed with 4% paraformaldehyde and embedded in paraffin. The samples were cut into 5 *μ*m sections and stained with hematoxylin-eosin (HE) and toluidine blue. The study observed the epidermal thickness and the number of mast cells in the sections.

### 2.9. Statistical Analysis

The study data were presented as the mean ± standard deviation and analyzed using SPSS software (version 24.0; IBM, Armonk, NY, USA). The data were compared between groups using one-way ANOVA and followed by multiple comparisons by Bonferroni's post hoc test. The value *P*  <  0.05 was considered significant.

## 3. Results

### 3.1. Body Weight and Skin Symptoms

**Figure 2:**
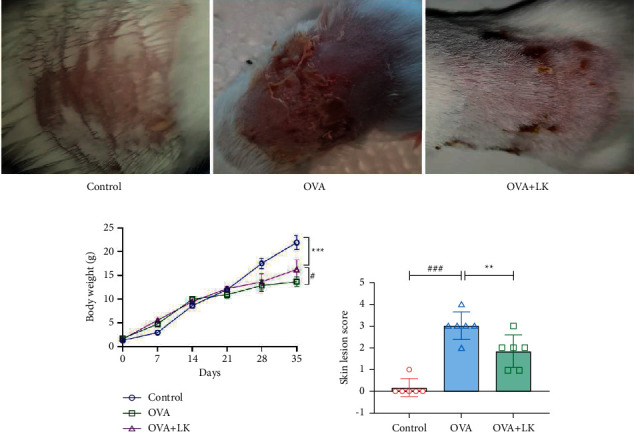
Body weight and skin lesion. The macroscopic characteristics of the skin (a). The body weight (b). Skin lesion score (c). Data are shown as the mean ± SD. ^#^*P* < 0.05 and ^###^*P* < 0.001 were for the comparison between the control group and the OVA group. ^∗∗^*P* < 0.01 and ^∗∗∗^*P* < 0.001 were for the comparison between the OVA + LK group and OVA group. one-way ANOVA with Bonferroni's post-hoc tests. OVA, ovalbumin. LK, probiotics mixture.

This study investigated the effects of probiotic mixtures on body weight and skin symptoms. The skin performance of juvenile mice in the OVA group showed dry skin, with obvious erythema, scaling, and scratch marks compared to the control group. However, the skin of juvenile mice in the mixed probiotics group was similar to that of the control group, with a significant reduction in erythema and scaling (Figure 2(a)) compared to the OVA group (Figure 2(a)). The study found that mice in the OVA group had a lower body weight than the control group, but when administered with a mixture of probiotics, their body weight significantly increased (Figure 2(b)). In addition, the OVA group had a higher skin lesion score than the control group, but the administration of probiotics resulted in a decrease in the skin lesion score (Figure 2(c)). Overall, the results suggest that the mixture of probiotics improved body weight and skin lesion scores in mice.

### 3.2. Skin Inflammation

**Figure 3:**
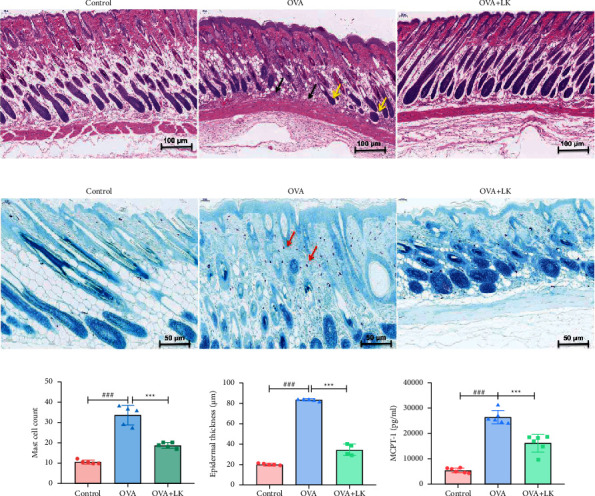
Skin tissue inflammation. HE staining, 100x (a). Toluidine blue staining, 200x (b). Mast cell count (c). Epidermal thickness (d). Concentrations of MCPT-1 (e). Data are shown as the mean ± SD. ^###^*P* < 0.001 was for the comparison between the control group and the OVA group. ^∗∗∗^*P* < 0.001 was for the comparison between the OVA + LK group and OVA group. one-way ANOVA with Bonferroni's post-hoc tests. OVA, ovalbumin. LK, probiotics mixture. MCPT-1, mast cell protease 1.

The inflammatory response is observed at the onset of AD in the skin [25].  Figure 3 illustrates the effects of OVA-induced dermatitis and probiotic mixture on this response. The results of HE staining indicated that the OVA group showed a significant thickening of the epidermis, infiltration of inflammatory cells in the dermis and subcutaneous tissues (black arrows), and degeneration of hair follicles (yellow arrows) compared to the control group. However, the mixed probiotics group showed improvement in these effects compared to the OVA group (Figure 3(a)).

Toluidine blue staining showed a significant increase in the number of mast cells and epidermal thickness in the OVA group compared to the control group. However, the mixed probiotics group exhibited a significant decrease in mast cells and epidermal thickness compared to the OVA group (Figures 3(b)–3(d)). Furthermore, the mixed probiotics group had reduced serum MCPT-1 concentrations compared to the OVA group (Figure 3(e)).

### 3.3. Serum Inflammatory Markers and Eosinophils

**Figure 4:**
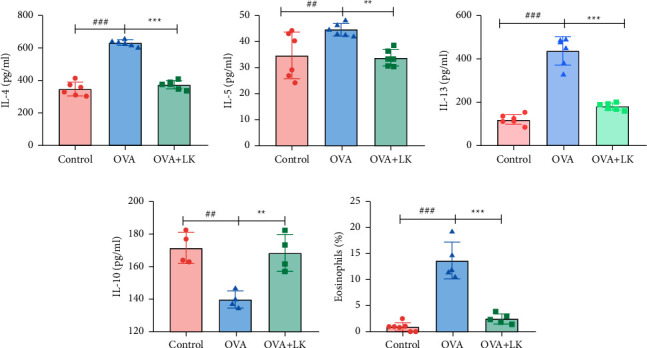
Serum inflammatory markers and eosinophils. Interleukin 4, IL-4 (a). Interleukin 5, IL-5 (b). Interleukin 13, IL-13 (c). Interleukin 10, IL-10 (d). Eosinophils (e). Data are shown as the mean ± SD. ^##^*P* < 0.01 and ^###^*P* < 0.001 were for the comparison between the control group and the OVA group. ^∗∗^*P* < 0.01 and ^∗∗∗^*P* < 0.001 were for the comparison between the OVA + LK group and OVA group. one-way ANOVA with Bonferroni's post-hoc tests. OVA, ovalbumin. LK, probiotics mixture. IL-4, Interleukin 4. IL-5, Interleukin 5. IL-13, Interleukin 13. IL-10, Interleukin 10.

The serum levels of IL-4, IL-5, and IL-13 were found to be significantly higher in the OVA group than in the control group. However, when administered with mixed probiotics, the serum levels of these cytokines were significantly reduced in comparison to the OVA group, showing an opposite trend to the anti-inflammatory cytokine IL-10. In addition, the study found that eosinophils were significantly increased in the OVA group compared to the control group, but were significantly reduced after being given mixed probiotics. These results suggest that mixed probiotics have the ability to inhibit OVA-induced inflammation (Figure 4).

### 3.4. OVA- Specific Serum IgE and Immunoglobulin G1 (IgG1)

**Figure 5:**
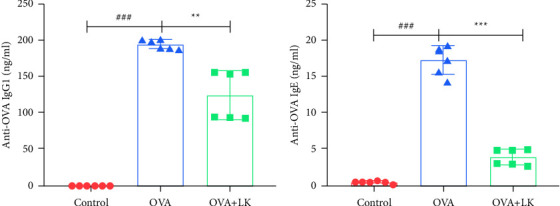
OVA-specific serum IgE and IgG1. Serum IgE antibodies (a). Serum IgG1 antibodies (b). Data are shown as the mean ± SD. ^###^*P* < 0.001 was for the comparison between the control group and the OVA group. ^∗∗^*P* < 0.01 and ^∗∗∗^*P* < 0.001 were for the comparison between the OVA + LK group and OVA group. one-way ANOVA with Bonferroni's post-hoc tests. OVA, ovalbumin. LK, probiotics mixture. IgE, immunoglobulin E. IgG1, immuneoglobulinG1.

This study analyzed the levels of IgE and IgG1 in the serum of three groups of mice. Results showed that the OVA group had higher levels of IgE and IgG1 than the control group. However, after treating the mice with probiotics, the serum concentrations of IgE and IgG1 significantly decreased compared with the OVA group (Figure 5). These findings suggest that probiotics may have a protective effect against allergies.

### 3.5. Effects of Mixed Probiotics on the Accumulation of Tregs and the Expression of PD-1 in SLNs

**Figure 6:**
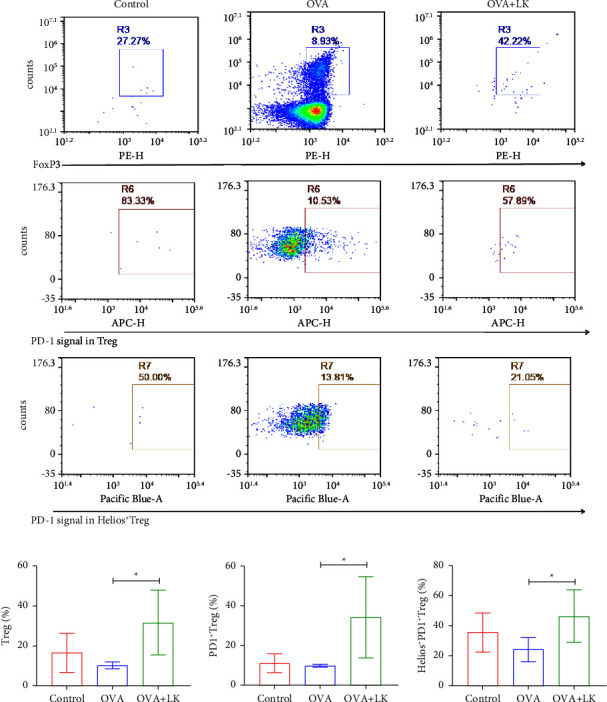
Mixed probiotics increased the accumulation of Tregs and enhanced PD-1 expression in SDLNs. Representative flow cytometry plots (a). The frequency of Tregs (b). The frequency of PD-1^+^Tregs (c). The frequency of Helios^+^PD-1^+^Tregs (d). Data are shown as the mean ± SD. ^∗^*P* < 0.05 was for the comparison between the OVA + LK group and OVA group. OVA, ovalbumin. LK, probiotics mixture. PD-1, programmed cell death 1. PD-L1, programmed cell death ligand 1.

Treg cells are a vital component of peripheral immune tolerance and play an important role in regulating tissue inflammation and maintaining the stability of the immune system, and the transcription factor Foxp3 plays a major immunosuppressive role [26, 27]. Therefore, we examined Tregs and their surface PD-1 expression levels in SLNs. In this study, Tregs and their surface PD-1 levels as well as Helios^+^PD-1^+^Tregs levels were significantly lower in the OVA group than in the control group. However, Tregs and their surface PD-1 levels were elevated in the mixed probiotics group compared to the OVA group. There were no changes in Helios^+^PD-1^+^Tregs in SLNs derived from the mixed probiotics group compared to the OVA group (Figure 6), which suggests that mixed probiotics increased the accumulation of Tregs and the expression of PD-1.

### 3.6. Effects of Mixed Probiotics on the Accumulation of CD103^+^DCs and PD-L1 Expression in SLNs

**Figure 7:**
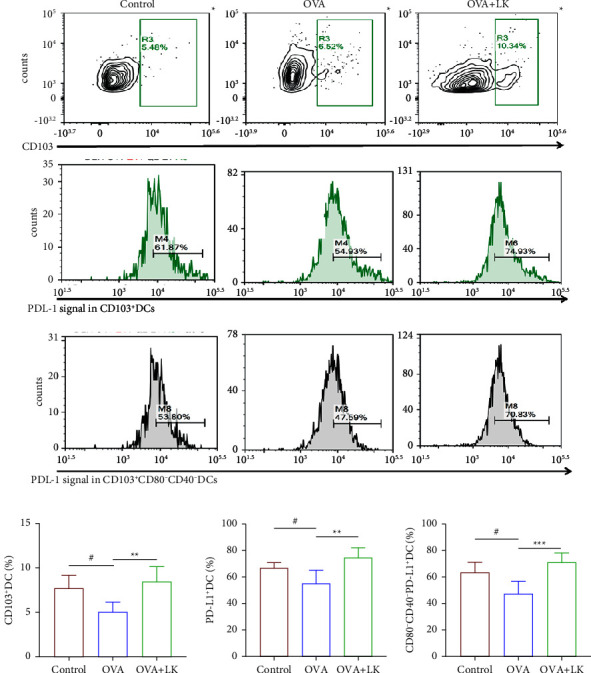
Mixed probiotics increased the accumulation of CD103^+^DCs and enhanced PD-L1 expression in SDLNs. Representative flow cytometry plots (a). The frequency of CD103^+^DC (b). The frequency of PD-L1^+^DC (c). The frequency of CD80^–^CD40^–^PD-L1^+^DC (d). Data are shown as the mean ± SD. ^#^*P* < 0.05 was for the comparison between the control group and the OVA group. ^∗∗^*P* < 0.01 and ^∗∗∗^*P* < 0.001 were for the comparison between the OVA + LK group and OVA group. OVA, ovalbumin. LK, probiotics mixture. DC, Dendritic cell.

CD103^+^DC is a specific DC subset that induces Foxp3^+^Tregs with immunosuppressive function in the gut. In this study, the level of expression of CD103^+^ DCs and their surface PD-L1 in SLNs was examined. It was found that the frequency of CD103^+^DCs in the OVA group was lower than that in the control group, whereas their frequency was significantly higher after mixed probiotics treatment compared with the OVA group. It was also found that the expression of PD-L1 on the surface of DC cells and CD103^+^CD80^−^CD40^−^DCs was significantly decreased in the OVA group compared to the control group. However, these changes were significantly improved with the mixed probiotics treatment (Figure 7). These results suggest that mixed probiotics promote DC accumulation in SLNs and increase the expression of PD-L1.

## 4. Discussion

Probiotics are healthy microbial strains that have antidiabetic, antiobesity, asthma relief, and food allergy effects [20, 21, 28, 29]. The present study was to investigate the effect of mixed probiotics on AD and their underlying mechanisms.

In this study, the mice were sensitized and challenged with OVA to establish an AD model as observed in a previous report [30]. Administration of a probiotic mixture to the mice inhibited the development of skin lesions and scratching behavior. Previous studies have reported that skin inflammation can intensify itchiness, and there is a correlation between the inflammatory response and scratching behavior [31]. In this study, the AD model mice showed significant infiltration of inflammatory cells, thickened epidermis, and elevated MCPT-1. However, these responses were significantly suppressed by the mixed probiotics. Mast cells, which are located in the mucosa, play a role in regulating immune inflammation [32], and also play a pathobiology role in type 2 inflammatory diseases of the airway [33]. Serum MCPT-1 levels can indicate the activation of systemic mast cells [34]. Overall, the study suggests that the mixed probiotics effectively reduced skin inflammation and scratching behavior. In this study, we also observed weight loss in juvenile AD mice, but the weight loss was improved after the use of a mixed probiotic. Consistent with our findings, a cohort study found that atopic dermatitis was associated with a shorter stature and lower weight in early childhood, although these associations were small [35]. Nowadays, a growing body of evidence supports the role of gut microbiota in influencing host appetite and food intake by regulating eating-related behavior. Supplementation of probiotics modifies gut microbiota that directly acts on molecules that regulate appetite and satiety [36]. Numerous studies have found that the supplementation of probiotics modulated hypothalamic control of food intake by improving leptin resistance in obesity [37]. We consider that in nonobese children with AD, weight loss occurs due to recurrent itching and itch-induced nocturnal sleep disturbances and decreased food intake. The use of probiotics alleviates itchy skin symptoms and regulates flora balance, thereby modulating the hypothalamus to increase food intake. However, more research is needed at a later stage to confirm probiotics and their relationship with appetite control in children with AD.

Helper T cell subsets participate in the immune response by producing specific cytokines. Among these, Th2 cells are crucial in the pathological process of AD [38]. The expression levels of IL-4, IL-13, and IL-5 are indicative of the activity of Th2 cells, which are central to humoral immunity and play a critical role in the pathogenesis of allergic inflammatory diseases [39]. The study observed a significant increase in Th2 cytokines in the serum of AD model mice, which were found to produce IL-4, IL-13, and IL-31, thus potentiating the barrier dysfunction and contributing to pruritus [40]. However, after mixed probiotics treatment, the Th2 cytokines were significantly decreased. In addition, the study found that IL-10, an immunosuppressive and anti-inflammatory cytokine, acts directly on Tregs and T-helper cells [41, 42]. Coomes' study found that IL-10 can reduce Th2 cell levels in allergic airway inflammation [43]. Eosinophils play a role in the pathogenesis of AD, allergic rhinitis, and asthma [44]. Furthermore, research has shown that the interaction between skin fibroblasts and eosinophils is crucial in provoking allergic inflammation in AD [45]. The diagnosis of AD can be aided by measuring the serum total IgE level and allergen-specific IgE. Common findings in AD include eczematous skin lesions and elevated serum IgE levels [46, 47]. Meanwhile, antigen-specific IgG may also play a role in AD [48]. It was found that IgG antibodies activate mast cells [49]. Antigen-specific IgE and IgG1 antibodies are systemic manifestations of Th2-type immune responses [50]. The study found that serum OVA-specific IgE and IgG1 were significantly increased in mice with AD but decreased after treatment with mixed probiotics, and these findings were consistent with previous research by Tamagawa-Mineoka et al. [51]. The results suggest that mixed probiotics can alleviate the Th2-type immune response in AD model mice and improve AD symptoms.

Treg cells play an important role in the maintenance and regulation of immune tolerance in the skin [52]. As previously mentioned, PD-1 and Helios play important roles in Treg cells' immune regulation [53, 54]. In this study, it was found that the Tregs and their surface expression levels of PD-1 in the SLNs were significantly decreased in AD model mice. However, the levels of Tregs, surface PD-1 of Tregs, and Helios^+^PD-1^+^Tregs were significantly increased after administration of mixed probiotics. This study further found that the expression of Treg and PD-1 was positively correlated with IL-10, suggesting that mixed probiotics may improve AD by increasing Th1, regulating Th1/Th2 balance, and increasing the amount of Treg. Tregs-induced immunosuppression requires PD-1 expression on autoreactive B cells and expression of two PD-1 ligands on Tregs. Tregs utilize PD-1 ligands to directly inhibit B cell activation and then inhibit antibody production [55]. It is shown that PD-1 reduces the cytotoxicity of T cells by inhibiting the production of cytotoxic effector molecules. T cell motility and the timing of interactions between DCs and target cells are also regulated by PD-1 [53]. Moreover, Helios is a transcription factor of activated Treg cells. The research found that CD103 is expressed at high levels on a subset of Helios^+^ Treg, and BrdU studies showed that, *in vivo*, Helios^+^Treg proliferated more than Helios^−^Treg. *In vitro* suppression assays show that Treg function correlates with the absolute number of Helios^+^ cells in culture [54].

Skin dendritic cells play an important role in the cross-presentation of pathogen-derived antigens and certain subtypes of DCs, such as CD103^+^DCs, and can modulate a hyperactive immune response and maintain immune homeostasis [56, 57]. As previously described, PD-L1 expressed on dendritic cells is a key mediator of germinal center effector cell tolerance and regulation, and initial activation of reactive CD4^+^T cells can be inhibited [58]. In this study, we analyzed the expression levels of CD103^+^DCs and their surface PD-L1 in the skin-draining lymph nodes and found that after the mixed probiotics intervention, the number of CD103^+^DCs in the SLNs of AD mice was significantly increased, and the surface PD-L1 of these DCs was significantly increased. Francisco et al. reported that PD-L1 could induce Tregs *in vitro*, and PD-L1 increased Foxp3 expression and enhanced the immunosuppressive ability of Tregs. Furthermore, by downregulating Akt, mTOR, and ERK2 while simultaneously upregulating PTEN, PD-L1 can convert naive CD4^+^T cells into Tregs [59]. Therefore, the PD-1/PD-L1 axis plays a crucial role in regulating Treg development and function.

Different DC subsets play distinct roles in the immune response [60]. This study further analyzed the relationship between the surface costimulatory molecules CD40, CD80, and PD-L1 levels of DCs, and found that the PD-L1 expression of CD103^+^CD80^−^CD40^−^DCs was significantly increased after mixed probiotics intervention. Vanherwegen et al. discovered that DCs with lower levels of costimulatory molecules, such as CD80, produced more IL-10 and higher levels of various cosuppressive molecules, such as PD-L1, and played an inhibitory regulatory role in the immune response [61]. Therefore, we speculate that the interaction of mixed probiotics and intestinal mucosa increases the level of PD-L1 on the surface of CD103^+^ DCs through the gut-skin axis and plays an immunoregulatory role through the PD-L1/PD-1 pathway to promote the differentiation of Tregs, thereby reducing the Th2-type immune response and the level of IgE. In this process, the CD103^+^CD80^−^CD40^−^DCs subset may play an important regulatory role.

In summary, mixed probiotics can reduce the number of mast cells involved in allergic and inflammatory reactions and the production of related inflammatory factors (MCPT-1); reduce the number of inflammatory cytokines (IL-4, IL-5, and IL-13) in serum and eosinophils in blood; and elevate the level of anti-inflammatory factor IL-10 in serum, thus reducing the inflammatory state of the skin tissues. In addition, mixed probiotics can modulate the immune response to AD via the PD-1/PD-L1 axis and reduce serum IgE and IgG1 levels, thereby improving the symptoms of atopic dermatitis. However, there may be large individual differences in how different people respond to probiotics. Atopic dermatitis is a complex disease with multiple pathogenic mechanisms, and probiotics have some potential in the treatment of atopic dermatitis, but more research is needed to determine their specific efficacy and indications. Moreover, the study uses a mouse model to investigate the effects of probiotics on AD. While mouse models are common in preclinical studies, their applicability to human AD can be limited. Differences in immune system responses and skin physiology between mice and humans may affect the translatability of these results. To further elucidate the PD-1/PD-L1 in the probiotic treatment of AD, future studies will involve the utilization of blocking antibodies and the acquisition of knockout mice to provide further validation of this pathway. In addition, 16S rRNA sequencing will be utilized to investigate the relationship between skin inflammation, PD-1/PD-L1, and gut microbiota.

## Data Availability

The data used to support the findings of the study are available from the corresponding author upon request.

## Abbreviations

AD: Atopic dermatitis
OVA: Ovalbumin
LK: Probiotics mixture
IgE: Immunoglobulin E
IL-10: Interleukin 10
IL-4: Interleukin 4
IL-5: Interleukin 5
IL-13: Interleukin 13
IL-10: Interleukin 10
MCPT-1: Mast cell protease 1.
